# Association between urinary arsenic and the prevalence of endometriosis in women in the United States

**DOI:** 10.3389/fpubh.2025.1525986

**Published:** 2025-03-24

**Authors:** Luyang Su, Yanan Ren, Ren Xu, Shixia Zhao, Weilan Liu, Cuiqiao Meng, Xuan Zhou, Zeqing Du

**Affiliations:** ^1^Physical Examination Center, Hebei General Hospital, Shijiazhuang, Hebei, China; ^2^Department of Obstetrics and Gynecology, Hebei General Hospital, Shijiazhuang, Hebei, China; ^3^Department of Obstetrics and Gynecology, Affiliated Hospital of Chengde Medical University, Chengde, Hebei, China; ^4^Department of Obstetrics and Gynecology, Second Hospital of Hebei Medical University, Shijiazhuang, Hebei, China

**Keywords:** endometriosis, arsenic, NHANES, gynecology, chronic inflammatory disease

## Abstract

**Background:**

Endometriosis affects up to 15% of women of reproductive age and can lead to various symptoms. More than 200 million people worldwide are at risk of higher than safe levels of arsenic exposure through drinking water. Studies investigating the relationship between arsenic and endometriosis are very limited and have yielded inconsistent results. This study aimed to explore the relationship between total urinary arsenic, arsenic species (Urinary arsenous acid, Urinary Arsenic acid, Urinary Arsenobetaine, Urinary Arsenocholine, Urinary Dimethylarsinic acid, Urinary Monomethylarsonic acid) and endometriosis.

**Methods:**

We utilized a nationally representative dataset from the National Health and Nutrition Examination Survey (NHANES) from 2003 to 2006. A total of 650 participants were included. We examined the association between total urinary arsenic and different arsenic species with endometriosis using weighted multivariate logistic regression models.

**Results:**

Urinary arsenous acid and urinary monomethylarsonic acid (MMA) were positively correlated with endometriosis (*p* < 0.05). After adjusting for potential confounding factors, the positive correlation of urinary MMA remained significant (OR: 1.317, 95%CI: 1.074–1.615). Subgroup analyses and interaction tests indicated that this association was not dependent.

**Conclusion:**

Our research underscores a significant positive association observed between factors urinary MMA and endometriosis. Future research is needed to elucidate the specific mechanisms behind this association.

## 1 Introduction

Endometriosis is characterized by the proliferation of endometrial-like tissue, including glandular cells and stroma, beyond the uterine cavity. The clinical presentation typically encompasses cyclical hemorrhage, dysmenorrhea, and dyspareunia (1). Epidemiological data suggests a prevalence rate of 10–15% in the female population of childbearing age (2). At present, the pathogenesis of endometriosis remains elusive. Proposed etiological hypotheses comprise retrograde menstruation, vascular or lymphatic dissemination, aberrant coelomic metaplasia, immune system dysregulation, and factors of cellular determinism, along with hereditary and environmental influences (2).

Arsenic is one of the most extensively studied elements in the field of metal toxicity, second only to lead (Pb) (3). Arsenic, a metalloid, is ubiquitously present in water, soil, and air, originating from both natural processes and human activities, existing in both inorganic and organic forms (4). Arsenic exposure through drinking water represents the primary mechanism of human contact, with over 200 million individuals worldwide facing the threat of encountering arsenic levels surpassing safety thresholds (5). Arsenic, a well-known metalloid, can cause endocrine disruption and reproductive disorders in women (6). Existing studies have linked arsenic exposure to ovarian dysfunction and infertility (7, 8). Exposure to high doses of arsenic during pregnancy increases the risk of miscarriage, fetal defects, and toxicity to the fetus (9, 10).

However, the relationship between arsenic exposure and endometriosis remains under-researched and is marked by contradictory findings (11, 12). Previous studies have not investigated the correlation between arsenic forms and endometriosis. Consequently, our research is designed to investigate the link between urinary total arsenic and different arsenic (urinary arsenous acid, urinary arsenic acid, urinary arsenobetaine, urinary arsenocholine, urinary dimethylarsinic acid, urinary monomethylarsonic acid) forms with endometriosis.

## 2 Methods

### 2.1 Data source

In this cross-sectional investigation, we employed data from the National Health and Nutrition Examination Survey (NHANES), which utilizes a sophisticated, multi-stage, stratified probability sampling method, ensuring samples that are representative of the national populace. The National Center for Health Statistics Institutional Review Board approved the data collection methods employed in the NHANES study, securing informed consent from all contributors. NHANES data are publicly available, enhancing transparency and facilitating scientific research across various fields. People can access extensive NHANES datasets, comprehensive operational manuals, consent forms, and periodical guides on the NHANES portal.^1^

### 2.2 Study participants

**Figure 1:**
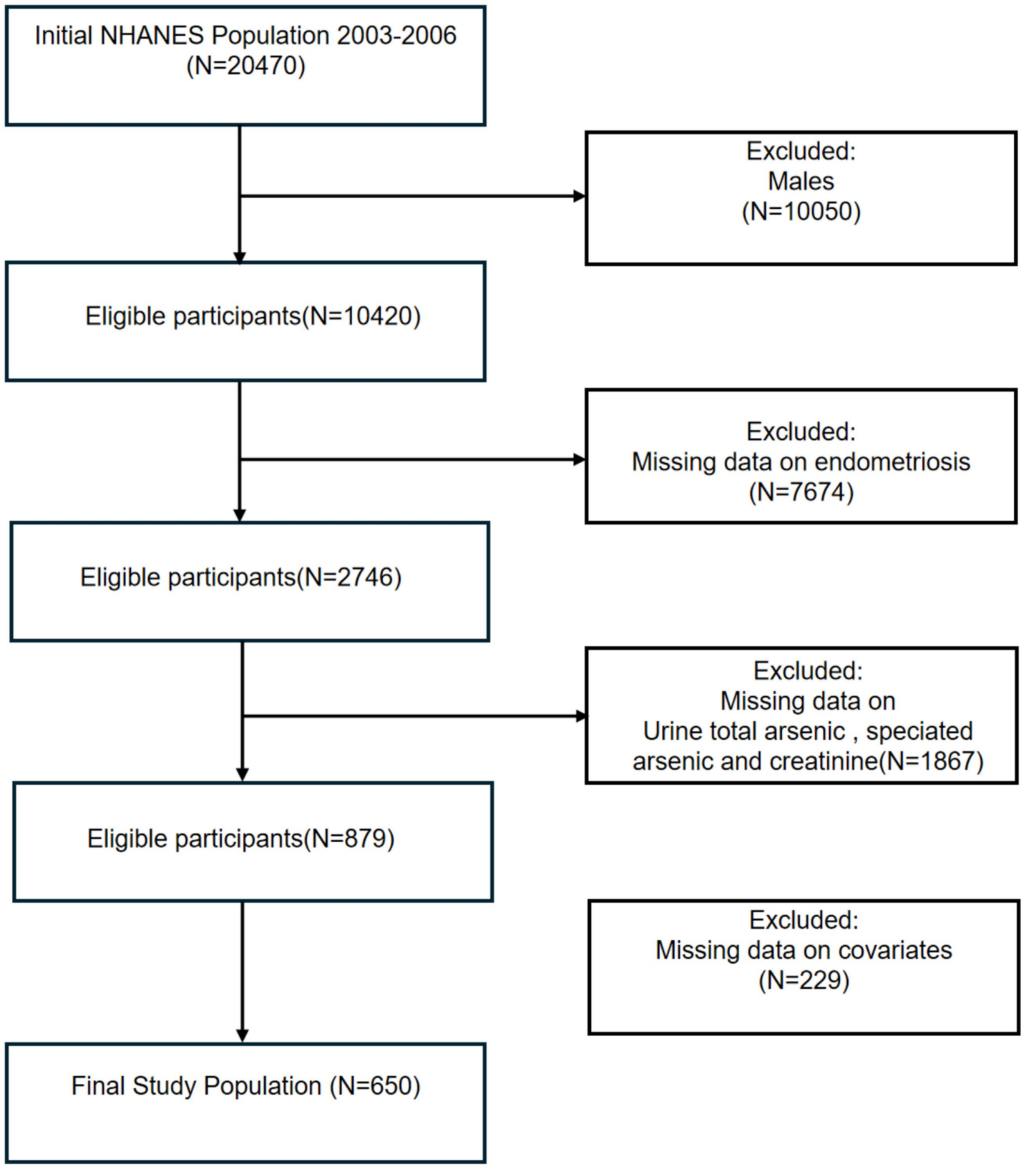
Flow chart of eligible participants’ selection.

This study analyzed data from two NHANES cycles (2003–2004 and 2005–2006), and all groups were initially included; the male group, those with missing information on endometriosis, those with urinary arsenic levels, and relevant covariates were excluded in turn, and 650 participants were included in the final analysis (Figure 1).

### 2.3 Urinary arsenic and creatinine measurement

This analysis used NHANES datasets for arsenic from 2003–2004 (L06UAS_C) and 2005–2006 (UAS_D), utilizing High-Performance Liquid Chromatography (HPLC) for species separation, followed by ICP-DRC-MS for detection of total urinary arsenic and species concentrations. The following arsenic species and their respective lower limit of detection (LLOD) were analyzed: Urinary total arsenic 0.74 μg/L, urinary arsenous acid 1.2 μg/L, urinary arsenic acid 1.0 μg/L, urinary arsenobetaine 0.4 μg/L, urinary arsenocholine 0.6 μg/L, urinary dimethylarsinic acid (DMA) 1.7 μg/L, and urinary monomethylarsonic acid (MMA) 0.9 μg/L. Concentrations below detection limits were substituted with LLOD/ √2 values. To correct for urine arsenic in urine cadmium concentration, we used urinary creatinine concentration (mg/dl) data from the same dataset.

### 2.4 Endometriosis status

The diagnosis of endometriosis was determined through a reproductive health questionnaire for women aged 20–54 years, asking, “Has a doctor or other health professional ever told you that you had endometriosis?”

### 2.5 Covariates assessment

Drawing from existing research and clinical insights, our analysis included the following covariates to minimize potential confounding effects. We gathered data on demographic aspects such as age, ethnicity (Non-Hispanic Black, Non-Hispanic White, Other), educational level (high school and below, above high school), marital status (married/living with a partner, widowed/divorced/separated, never married), and the poverty income ratio (PIR). The Body Mass Index (BMI), determined by the individual’s mass in kilograms divided by the square of their height in meters, was classified into either normal or underweight (BMI < 25 kg/m^2^) and overweight or obese (BMI ≥ 25 kg/m^2^). We queried participants about their smoking status with the question, “Have you smoked at least 100 cigarettes in your lifetime?” and measured alcohol consumption by asking, “In the past 12 months, have you consumed 4 to 5 or more drinks per day?” Histories of diabetes and hypertension were acquired from self-reports in our health survey questionnaire. Additional data on age at menarche, fertility status were gleaned from the reproductive health questionnaire.

### 2.6 Statistical analysis

To delineate the variations in baseline characteristics between individuals with and without endometriosis, categorical variables were represented as weighted proportions, and continuous variables as weighted means with standard deviation or weighted medians with interquartile ranges. Prior to conducting logistic regression analyses, we evaluated the correlations between different arsenic species using Spearman’s correlation coefficients. To avoid potential multicollinearity issues, we examined the variance inflation factors for all arsenic species. The variance inflation factors values were all below 5, indicating no significant multicollinearity among the arsenic species. Based on these analyses, we conducted separate logistic regression models for each arsenic species to avoid the potential confounding effects of inter-species correlations. Weighted logistic regression analyses, both univariate and multivariate, were applied to gauge the relationship between six arsenic species and endometriosis. Model 1 did not control for any covariates, Model 2 was adjusted for age and ethnicity, and Model 3 further controlled for marital status, educational attainment, smoking status, BMI, PIR, menarche onset, alcohol use, hypertension, diabetes, fertility status and creatinine levels in urine. The adoption of restricted cubic splines (RCS) served to address the nonlinear relationship between MMA and endometriosis. Finally, statistical *p* for interaction among covariates and endometriosis prevalence rate was assessed, and subgroup analyses were performed to enhance understanding of these findings. In addition, age-stratified analysis using 40 years as the cutoff point was conducted, taking into account both the natural course of endometriosis and the potential cumulative effects of arsenic exposure. Besides, the multiple imputation (MI) approaches were applied by us to fill in the missing values that exist in the data through the “MICE” package of the R software. R and RStudio (version 4.3.0) facilitated all statistical computations, with *p*-values below 0.05 denoting statistical significance.

## 3 Results

### 3.1 Characteristics of the participants

The baseline characteristics and weighted estimates of the 650 participants were detailed in Table 1. Out of the total participants, 44 were afflicted with endometriosis, while 606 remained unaffected. Substantial variations (*p* < 0.05) were evident between the endometriosis and non-endometriosis cohorts concerning age, BMI, ethnicity. There were no significant differences (*p* > 0.05) in menarche age, PIR, marital status, smoking status, education level. The distribution of laboratory measurement results among participants was displayed in Table 2, with MMA being the only indicator significantly associated with endometriosis. Individuals with endometriosis were more likely to have urinary MMA concentrations at or above the detection limit.

**Table 1:** Characteristics of NHANES participants included in the analysis.

Characteristics	Non-endometriosis (*N* = 606)	Endometriosis (*N* = 44)	*p* value
Age (years)	39.20 (9.42)	41.08 (6.40)	0.049
Age at menarche (years)			0.710
<12	143 (22.7%)	11 (17.7%)	
12–13	301 (50.1%)	22 (54.8%)	
≥14	162 (27.2%)	11 (27.5%)	
PIR	2.96 (1.59)	3.27 (1.65)	0.249
BMI (kg/m^2^)			0.039
<25	173 (33.6%)	20 (52.1%)	
≥25	433 (66.4%)	24 (47.9%)	
Smoked ≥100cigarettes			0.971
Yes	217 (40.6%)	14 (41.0%)	
No	389 (59.4%)	30 (59.0%)	
Race/ethnicity			0.003
Non-Hispanic White	263 (66.4%)	32 (89.2%)	
Non-Hispanic Black	162 (15.7%)	7 (5.2%)	
Other	181 (18.0%)	5 (5.7%)	
Marital status			0.233
Married/Living with partner	434 (75.9%)	28 (64.0%)	
Never married	79 (15.0%)	5 (24.9%)	
Widowed/Divorced/Separated	93 (9.1%)	11 (11.1%)	
Education level			0.153
High school and below	153 (18.4%)	4 (8.1%)	
Above high school	453 (81.6%)	40 (91.9%)	
Hypertension			0.801
Yes	116 (20.5%)	11 (22.6%)	
No	490 (79.5%)	33 (77.4%)	
Diabetes			0.077
Yes	38 (6.3%)	0 (0.0%)	
No	568 (93.7%)	44 (100.0%)	
Alcohol intake			0.822
Yes	359 (65.8%)	26 (63.8%)	
No	247 (34.2%)	18 (36.2%)	
Fertility status			0.420
Nulliparous	39 (7.5%)	5 (11.3%)	
≥one birth	567 (92.5%)	39 (88.7%)	

Continuous variables were presented as median (interquartile range) or mean (standard deviation). Categorical variables were presented as frequency (percentage).

**Table 2:** Laboratory measures of NHANES participants included in the analysis.

Characteristics	Non-endometriosis (*N* = 606)	Endometriosis (*N* = 44)	*p-*value
Creatinine, urine (mg/dL)	103.00 (64.00, 161.65)	108.96 (40.04, 146.53)	0.540
Urinary arsenic, total (μg/L)	7.96 (3.79, 17.19)	8.22 (2.42, 13.89)	0.192
Urinary arsenous acid (μg/L)			0.778
At or above detection limit	15 (2.2%)	1 (3.1%)	
Below lower detection limit	591 (97.8%)	43 (96.9%)	
Urinary arsenic acid (μg/L)			0.867
At or above detection limit	38 (9.5%)	4 (8.8%)	
Below lower detection limit	568 (90.5%)	40 (91.2%)	
Urinary arsenobetaine (μg/L)			0.775
At or above detection limit	432 (68.8%)	32 (71.0%)	
Below lower detection limit	174 (31.2%)	12 (29.0%)	
Urinary arsenocholine (μg/L)			0.958
At or above detection limit	12 (2.9%)	1 (3.1%)	
Below lower detection limit	594 (97.1%)	43 (96.9%)	
Urinary DMA (μg/L)			0.487
At or above detection limit	515 (83.8%)	36 (78.6%)	
Below lower detection limit	91 (16.2%)	8 (21.4%)	
Urinary MMA (μg/L)			0.010
At or above detection limit	168 (30.1%)	21 (55.1%)	
Below lower detection limit	438 (69.9%)	23 (44.9%)	

Continuous variables were presented as median (interquartile range) or mean (standard deviation). Categorical variables were presented as frequency (percentage). DMA, dimethylarsinic acid; MMA, monomethylarsonic acid.

### 3.2 Weighted logistic regression and subgroup analyses

Weighted univariate and multivariate logistic regression analyses were utilized to examine the correlation between six types of arsenic and endometriosis. As depicted in Table 3, Model 1 did not adjust for covariates, Model 2 adjusted for age and race, and Model 3 was subjected to further adjustments incorporating marital status, education level, smoking status, BMI, PIR, age at menarche, alcohol use, hypertension, diabetes, fertility status and urine creatinine levels. Associations were reported using odds ratios (OR) with 95% confidence intervals (CI). Higher concentrations of arsenous acid and MMA in urine were positively correlated with endometriosis (*p* < 0.05). Although the positive correlation of arsenous acid weakened after adjusting for potential confounding factors, MMA remained significantly correlated with endometriosis (MMA: OR: 1.317, 95%CI: 1.074–1.615).

**Table 3:** Unadjusted and adjusted odds ratios (OR) and 95% confidence intervals (CI) of the association between arsenic and endometriosis NHANES 2003–2006.

Speciated urinary arsenic	OR (95% CI) *p*		
	Model 1	Model 2	Model 3
Urinary arsenous acid (μg/L)	1.974 (1.136–3.430) 0.018	1.898 (1.072–3.362) 0.029	1.741 (0.961–3.154) 0.065
Urinary arsenic acid (μg/L)	0.958 (0.612–1.501) 0.847	0.905 (0.536–1.526) 0.697	0.842 (0.443–1.602) 0.576
Urinary arsenobetaine (μg/L)	0.997 (0.989–1.005) 0.405	0.997 (0.990–1.004) 0.356	0.997 (0.992–1.003) 0.313
Urinary arsenocholine (μg/L)	0.317 (0.009–11.461) 0.031	0.242 (0.005–11.500) 0.457	0.363 (0.018–7.463) 0.484
Urinary dimethylarsinic acid (DMA) (μg/L)	1.000 (0.952–1.050) 0.992	1.013 (0.970–1.058) 0.550	1.005 (0.961–1.051) 0.825
Urinary monomethylarsonic acid (MMA) (μg/L)	1.341 (1.127–1.595) 0.002	1.356 (1.106–1.663) 0.005	1.317 (1.074–1.615) 0.012

Model 1 did not control for any covariates, Model 2 was adjusted for age and ethnicity, and Model 3 further controlled for marital status, educational attainment, smoking status, BMI, PIR, menarche onset, alcohol use, hypertension, diabetes, fertility status and creatinine levels in urine.

**Figure 2:**
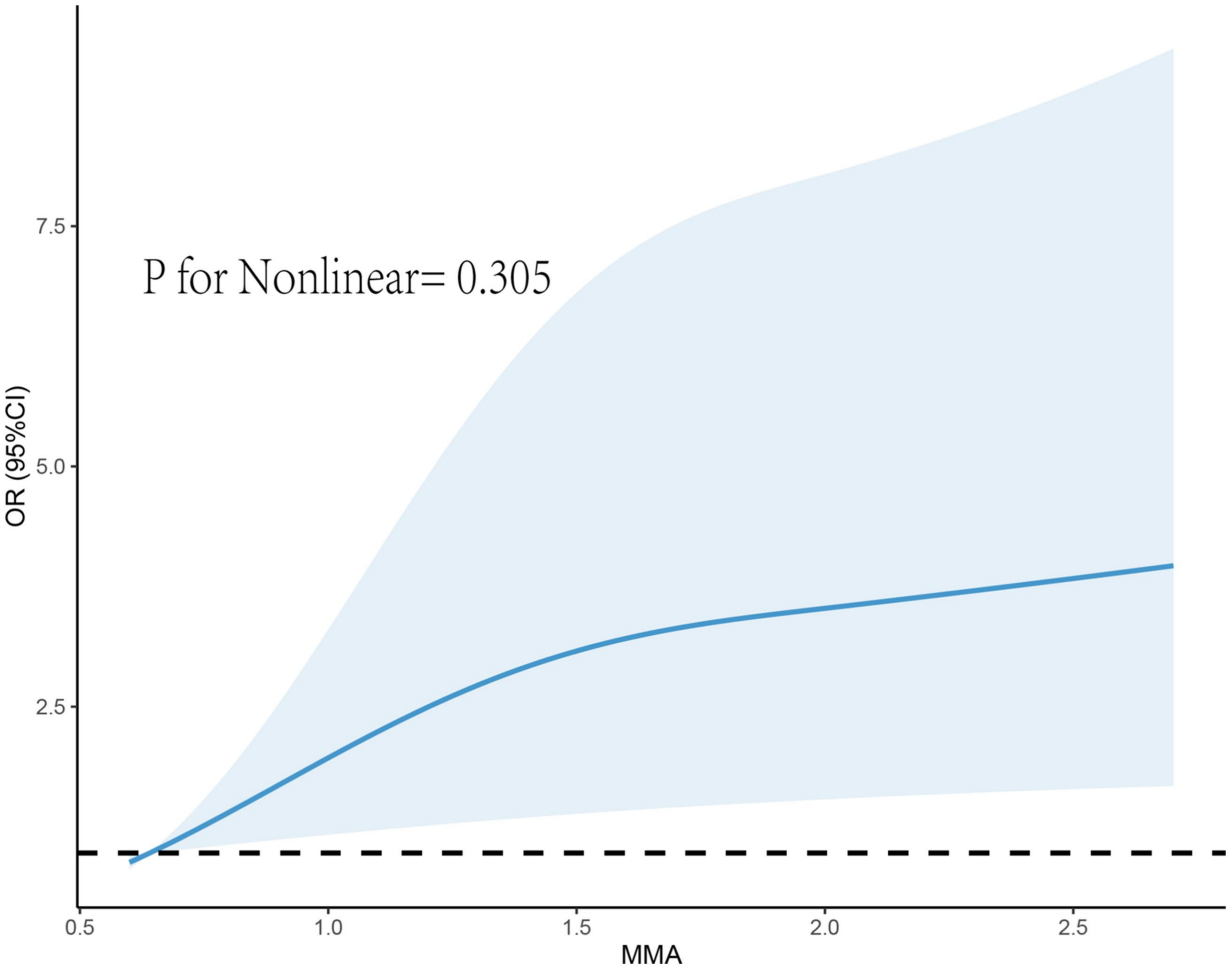
Restricted cubic spline plot of the association between monomethylarsonic acid (MMA) and endometriosis.

**Figure 3:**
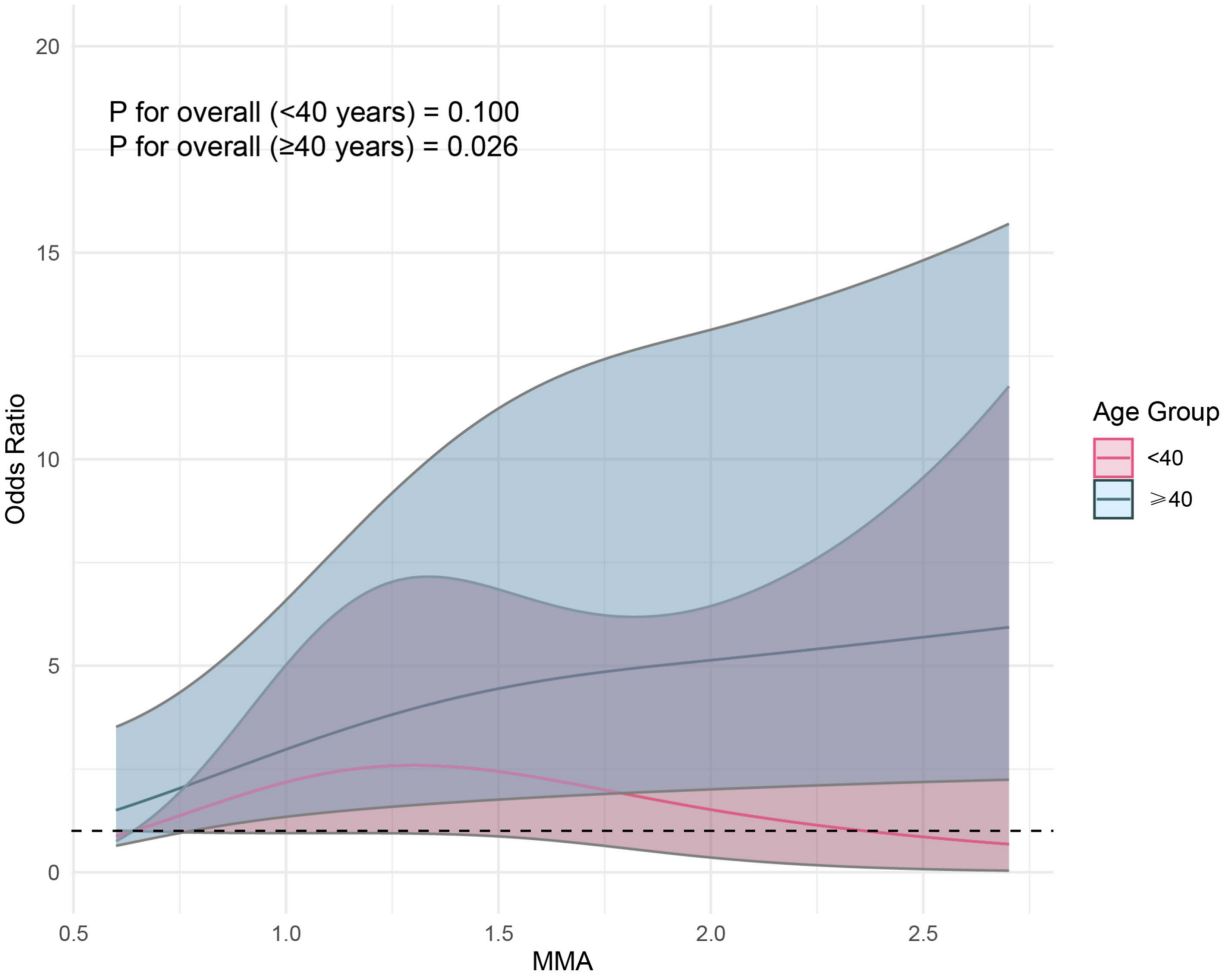
Restricted cubic spline plot of the association between monomethylarsonic acid (MMA) and endometriosis stratified by age.

After adjusting for covariates in Model 3, although RCS did not reveal a nonlinear relationship between MMA and endometriosis, the prevalence rate of endometriosis seemed to increase with higher exposure levels (Figure 2). Furthermore, we found that the correlation between MMA and endometriosis was not significant in individuals under 40 years old but was significant in those over 40 (Figure 3), possibly related to long-term arsenic exposure. The sensitivity analysis results after multiple interpolation indicate that they were still robust (Supplementary Table S1).

**Figure 4:**
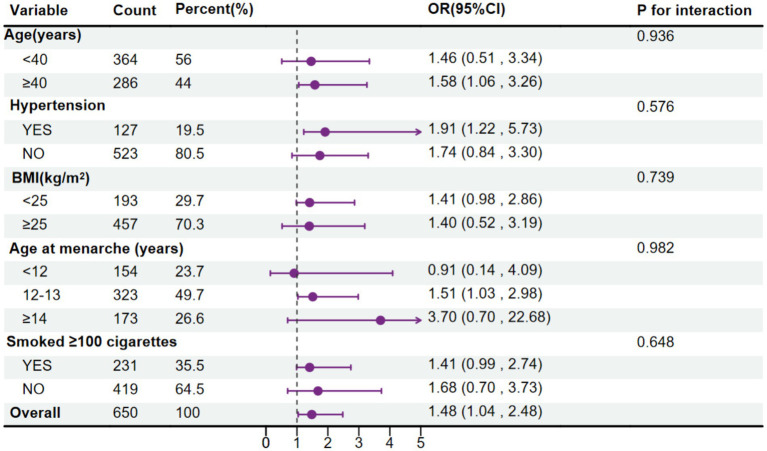
Forest plot for the association between urinary monomethylarsonic acid (MMA) and endometriosis, stratified by age, body mass index (BMI), age at menarche, hypertension and smoking status.

As illustrated in Figure 4, when observing the association between MMA and endometriosis, grouped by age, marital status, education level, BMI, age at menarche, smoking status, and race, no interaction of the related covariates was observed in this association (*p* for interaction > 0.05), indicating that the correlation between MMA and endometriosis does not vary due to age, BMI, age at menarche, smoking status, and hypertension.

## 4 Discussion

In our nationally representative study of American women, we found a correlation between urinary MMA and self-reported physician-diagnosed endometriosis, after adjusting for potential confounders. As far as we are aware, this serves as the inaugural large-scale, nationally representative inquiry into the relationship between total urinary arsenic, arsenic species, and endometriosis, unveiling a positive correlation between urinary MMA and endometriosis. Studies on arsenic and endometriosis are limited and inconsistent; a previous cohort study involving 473 women did not observe a correlation between urinary arsenic and endometriosis (12). In China, a recent study on the association between exposure to various toxic metals and endometriosis found that the concentration of arsenic in blood or follicular fluid contributed the most, reporting a positive correlation with endometriosis (11).

As an estrogen-dependent chronic inflammatory disease, endometriosis has the highest prevalence rate between the ages of 25–35 (13). Estrogen plays a crucial role in the disease’s pathogenic mechanism; increased local production of estrogen fosters the survival, implantation, and proliferation of ectopic endometrial tissue (14). The relationship between arsenic and estrogen is still controversial. A study based on adolescents showed a positive correlation between total arsenic, its metabolites, and estradiol (14), while two experimental studies indicated a significant decrease in estradiol levels after arsenic exposure (15, 16). Arsenic interacts with the ligand-binding domain of estrogen receptor-alpha (ERα) with high affinity, exhibiting potent estrogenic activity *in vitro* and *in vivo* (17). Moreover, arsenic, characterized by its properties as a metalloid with both metallic and nonmetallic attributes, predominantly manifests its toxic effects through oxidative stress, widely acknowledged as the principal mechanism of arsenic toxicity (18, 19). Reactive oxygen species (ROS) production can alter redox balance, ultimately leading to reproductive abnormalities such as miscarriage, endometriosis, and polycystic ovary syndrome (20, 21). Studies have found that antioxidant defense mechanisms in the uterus of mice, Including enzymes like peroxidase (GPx), superoxide dismutase (SOD), and catalase (CAT), are reduced by 2.5, 2.6, and 2.9 times, respectively, in arsenic-exposed groups (16). MMA is a major component of arsenic metabolites, with up to 11% of total urinary arsenic excreted as MMA (22). It is one of the most toxic forms of arsenic in the human body (22, 23), possibly due to its higher cellular uptake rate (24) and high affinity for thiol (-SH) groups (22). Although the toxicological characteristics of arsenic metabolites vary in the body, the toxicity phenotype of MMA is mostly associated with ROS production (3). Arsenic exposure also leads to an increase in pro-inflammatory cytokines such as IL-6, IL-10, and TNF-*α*, which are closely related to inflammatory responses (25). The inflammatory pathway is also involved in the mechanism of endometriosis formation (26). The complexity of arsenic toxicity is manifested through its multifaceted nature (27). Nevertheless, owing to the scarcity of specific research examining arsenic exposure in relation to endometriosis, further experiments are warranted to elucidate the involved mechanisms.

This study has the following limitations: Firstly, as it is a cross-sectional study design, potential reverse causation cannot be ruled out. The study measures current arsenic levels but cannot account for the time elapsed between endometriosis diagnosis and arsenic measurement. Secondly, although urinary arsenic concentration is commonly used as a typical biomarker for arsenic exposure, the rapid metabolism in humans means that data from a single urine sample may limit our ability to accurately assess the average level of arsenic exposure. Additionally, the distribution of arsenic forms differs between blood and urine, with blood arsenic potentially providing a more accurate reflection of exposure levels in target tissues and organs (28). Furthermore, our analytical approach, which focused on single urinary arsenic measurements, represents a methodological limitation. More sophisticated mixed-effects analyses, such as weighted quantile sum regression or Bayesian kernel machine regression, could provide deeper insights into the complex relationships between multiple arsenic species and endometriosis risk. Lastly, most of the data used were collected through interviews or self-reported questionnaires, which could be subject to recall and reporting biases.

## 5 Conclusion

In conclusion, this investigation establishes the initial evidence of a direct link between urinary MMA and the prevalence of endometriosis. Considering the significant prevalence of endometriosis and the pervasive exposure to arsenic in the general populace, it is vital to pursue further experimental studies to validate our findings and elucidate the specific mechanisms involved.

## Data Availability

The datasets presented in this study can be found in online repositories. The names of the repository/repositories and accession number(s) can be found at: all the data analyzed in our study can be obtained from NHANES database.

## Footnotes

^1^ https://www.cdc.gov/nchs/nhanes
