# Supplementary material for: Association between urinary arsenic and the prevalence of endometriosis in women in the United States
Source: Front Public Health. 2025 Mar 24;13:1525986. doi: 10.3389/fpubh.2025.1525986 (PMC11973268; doi:10.3389/fpubh.2025.1525986)
Supplement: Supplementary file 1 [file Table_1.docx]

**Table S1** Unadjusted and adjusted odds ratios (OR) and 95% confidence intervals (CI) of the association between arsenic and endometriosis after multiple imputation

| Speciated urinary arsenic | OR (95% CI) *P* | | |
| --- | --- | --- | --- |
|  | Model 1 | Model 2 | Model 3 |
| Urinary arsenous acid (μg/L) | 2.147  (1.219-3.780) 0.010 | 2.068  (1.132-3.781) 0.020 | 2.127  (0.987-4.010) 0.060 |
| Urinary arsenic acid (μg/L) | 0.987  (0.662-1.472) 0.949 | 0.944  (0.613-1.456) 0.788 | 0.876  (0.512-1.498) 0.601 |
| Urinary arsenobetaine (μg/L) | 0.990  (0.965-1.014) 0.391 | 0.992  (0.976-1.008) 0.311 | 0.993  (0.979-1.008) 0.354 |
| Urinary arsenocholine (μg/L) | 0.237  (0.002-23.057) 0.525 | 0.193  (0.002-19.542) 0.470 | 0.752  (0.021-27.191) 0.865 |
| Urinary dimethylarsinic acid (DMA) (μg/L) | 0.996  (0.936-1.060) 0.892 | 1.007  (0.954-1.064) 0.790 | 1.009  (0.964-1.057) 0.666 |
| Urinary monomethylarsonic acid (MMA) (μg/L) | 1.435  (1.123-1.834) 0.005 | 1.429  (1.092-1.869) 0.011 | 1.563  (1.126-2.170) 0.011 |

Model 1 did not control for any covariates, Model 2 was adjusted for age and ethnicity, and Model 3 further controlled for marital status, educational attainment, smoking status, BMI, PIR, menarche onset, alcohol use, hypertension, diabetes, fertility status and creatinine levels in urine.
